# Self‐concept, creativity and developmental dyslexia in university students: Effects of age of assessment

**DOI:** 10.1002/dys.1722

**Published:** 2022-07-11

**Authors:** Nicola Brunswick, Serena Bargary

**Affiliations:** ^1^ Department of Psychology Middlesex University London UK; ^2^ Priory Hospital Roehampton London UK

**Keywords:** creativity, dyslexia, estimated intelligence, self‐efficacy, self‐esteem

## Abstract

Educational experiences often influence self‐concept. Thus, readers with dyslexia can have low self‐esteem and self‐efficacy, and perceive themselves as less intelligent than their peers. They may develop creativity to succeed despite their difficulties but findings are inconsistent and rarely consider the effect of age of assessment on self‐perception. This study included 145 university students (*M*age = 24.43 years), 72 with dyslexia; of these, 53% had been assessed in childhood (*M*age = 11.89 years), 47% in adulthood (*M*age = 27.38 years). A survey assessed self‐esteem, self‐efficacy, creativity and estimated intelligence. Students with dyslexia reported lower levels of self‐esteem, self‐efficacy and estimated intelligence. When assessment age was considered, those assessed early displayed lower self‐esteem and self‐efficacy but no difference in estimated intelligence. Those assessed late displayed lower estimated intelligence and self‐esteem but no difference in self‐efficacy. Findings highlight the importance of providing psychological support to students with dyslexia to enhance their self‐perceptions.

## 1 INTRODUCTION

Society typically places high value on educational achievements which are partly predicated on the development of fluency in reading and spelling. Yet for a significant number of individuals literacy skills do not develop smoothly and educational achievements do not come easily. Approximately 10% of people have dyslexia (All‐Party Parliamentary Group for Dyslexia and Other Specific Learning Difficulties, 2019a), a neurodevelopmental difference characterized by difficulty with accurate and fluent reading and spelling alongside difficulties with phonological processing, verbal memory, organization, attention, verbal communication and processing speed (Rose, 2009). These difficulties impact on the individual's ability to meet the expectations of a literacy‐based education system.

Educational experiences have been shown to influence self‐perception and self‐concept, that is, individuals' beliefs about themselves (Lawrence, 1996; Marsh & Craven, 2006). The concept of the self is learned and regarded by many as multidimensional (e.g., Arens & Jansen, 2016; Marsh & Martin, 2011) so individuals can appraise themselves as being talented in one domain whilst holding a lower self‐view in another (Marsh & O'Mara, 2008). Within this formation of identity lie self‐esteem and self‐efficacy. The former reflects a feeling of “worthiness” (“I am happy being the way I am”; Brown & Marshall, 2006; Zeigler‐Hill, 2013) and may reflect one's perception of the self in general or in specific areas such as the academic, physical or social self. The latter is an appraisal of one's ability to influence events (“If I am in a difficult situation I can usually think of a way out”; Bandura, 2010) and may also be context‐specific.

These components underpin emotional well‐being, and both have been identified as being vulnerable in many readers with dyslexia, particularly those for whom their dyslexia was missed or poorly supported at school leading to repeated academic failure (Burden, 2008; Carroll & Iles, 2006; Glazzard, 2010; Morgan, Fuchs, Compton, Cordray, & Fuchs, 2008). Several studies report that children with dyslexia who attend mainstream schools have lower self‐esteem than children who attend specialist dyslexia schools (Humphrey & Mullins, 2002; Jones & Heskin, 2010; Nalavany, Carawan, & Brown, 2011), and often make negative statements regarding their academic ability in comparison to their peers (Humphrey & Mullins, 2002). Feelings of isolation, stupidity and “being different” are key experiences for children with dyslexia (Glazzard, 2010; Ingesson, 2007; Leitão et al., 2017) many of whom report being bullied and teased (Glazzard, 2010; Hellendoorn & Ruijssenaars, 2000; Singer, 2005). The UK's All‐Party Parliamentary Group for Dyslexia and Other Specific Learning Difficulties (2019a) survey found that 95% of parents of children with dyslexia said that their children experienced frustration because of their [poorly supported] dyslexia, 88% said that their children had poor self‐esteem, 84% said that they suffered from anxiety, and 78% said that their children felt embarrassed. When children with dyslexia experience success they are more likely to attribute this to external factors than internal ones such as their own intelligence, emphasizing their low levels of self‐efficacy and “learned helplessness” (Lithari, 2019).

While most research into the social and emotional effects of dyslexia has focussed on children, less attention has been paid to the period of early adulthood as individuals move beyond compulsory schooling into higher education (see McArthur, Filardi, Francis, Boyes, & Badcock, 2020). Numbers of students with dyslexia enrolling at university have increased in recent years (Olofsson, Taube, & Ahl, 2015) although these are still fairly low at around 3–5% of all UK students (Higher Education Statistics Agency, 2014; Richardson, 2015). Even though these individuals have achieved qualifications necessary to secure a university place, the academic demands of higher education render them vulnerable to ongoing anxiety, frustration and low self‐efficacy (Doiku‐Avlidou, 2015; Mcllroy, Poole, Ursavas, & Moriarty, 2015; Mortimore & Crozier, 2006) although these feelings may diminish over time (Stampoltzis, Antonopoulou, Zenakou, & Kouvava, 2010). Lithari (2019) writes about young adults “repairing” their fractured self‐perception only once they have left the academic pressures of compulsory education. This may be achieved through the recognition of real‐world (non‐literacy‐based) achievements (Doiku‐Avlidou, 2015; McNulty, 2003), or the development of adaptive strategies such as determination, resilience and creativity (described as “learnt creativity”: Gerber, Ginsberg, & Reiff, 1992; see also Burns, Poikkeus, & Aro, 2013; Firth, Frydenberg, Steeg, & Bond, 2013).

Some studies report that compared with readers without dyslexia, those with dyslexia score higher on measures of creativity and innovative thinking (Kapoula et al., 2016; Pąchalska, Bogdanowicz, Tomaszewska, Łockiewicz, & Bogdanowicz, 2009; Tafti, Hameedy, & Baghal, 2009), possibly as a result of their preference for visual representations and ability to generate novel solutions to problems (Bacon & Bennett, 2013; Cockcroft & Hartgill, 2004). Others, however, using a variety of definitions and measures of creativity, report no differences (Alves & Nakano, 2014; Łockiewicz, Bogdanowicz, & Bogdanowicz, 2014; Mourgues, Preiss, & Grigorenko, 2014; Ritchie, Luciano, Hansell, Wright, & Bates, 2013). Two recent meta‐analyses of dyslexia and creativity have helped to resolve some of this inconsistency: both found that while readers with dyslexia as a group are no more creative than those without dyslexia, adults with dyslexia (but not children or adolescents) are significantly more creative than adults without dyslexia (Erbeli, Peng, & Rice, 2022; Majeed, Hartanto, & Tan, 2021). Of course, it may not be the *measured* ability itself that is important: readers with dyslexia are widely believed to be creative (anecdotal evidence abounds of dyslexic designers, architects and artists – see Brunswick, 2009), so it may be that *perception* of one's own creativity will be evident amongst these readers irrespective of their actual ability. This may feed into a heightened sense of competence and self‐worth (see de Beer, Engels, Heerkens, & van der Klink, 2014 for a review).

An additional factor that may affect the self‐esteem and self‐efficacy of adults with dyslexia is the age at which their dyslexia was identified. A small number of qualitative studies has argued that early identification optimizes well‐being and educational achievement. However, these studies generally include interviews with between 4 and 20 people (Armstrong & Humphrey, 2009; Gibson & Kendall, 2010; Pitt & Soni, 2017; Rowan, 2014; Stampoltzis & Polychronopoulou, 2009), thus limiting their generalizability. By contrast, Ingesson (2007) interviewed 75 adolescents and young adults with dyslexia, and found that younger adolescents (7–13 years) were particularly vulnerable to low self‐esteem as a result of their dyslexia making them feel “different, inferior and stupid” (p. 49) although these feelings lessened over time. No direct comparison was made between these adolescents with dyslexia and their non‐dyslexic peers, or between those who were assessed early and those who were assessed later, therefore it is difficult to tease apart the effects of age and dyslexia on reported self‐esteem and estimated intelligence. However, it is easy to see how repeated negative experiences within education might affect the self‐concept of individuals with dyslexia, leading them to doubt their own intelligence and ability.

The present study, therefore, was designed to explore the self‐perceptions of university students with and without dyslexia – their beliefs regarding self‐esteem, self‐efficacy, intelligence and creativity – to see how the perceptions of those with dyslexia may have been influenced by their experience of being assessed in childhood or adulthood. It was hypothesized that students with dyslexia would score lower than their non‐dyslexic peers for self‐esteem, self‐efficacy and estimated IQ, but that they would rate their creativity higher. It was further hypothesized that an earlier assessment would offer some protection (in terms of self‐esteem, self‐efficacy and estimated IQ) not afforded to those who were assessed later.

## 2 MATERIALS AND METHODS

An anonymous online survey was employed as readers with dyslexia are often difficult to access, and we wanted to recruit as large a sample as possible (see also Nalavany & Carawan, 2012). There was one quasi‐independent variable (reading ability) with either two levels (students with or without dyslexia) or three levels (students without dyslexia, and students with dyslexia who were either assessed in childhood or adulthood). Dependent variables were estimated intelligence, self‐esteem, self‐efficacy and creativity.

### 2.1 Participants

Participants were recruited through social‐media posts on Facebook and Twitter inviting people to participate in a survey about self‐concept and creativity in students in higher education. Students with dyslexia were targeted specifically via posts to private dyslexia help and support groups on Facebook, with the permission of the group administrators, and via public Tweets using #dyslexic. The survey was also advertised on Prolific (www.prolific.co), requesting participants who were English speakers, currently studying in the UK and who had been assessed and identified as having dyslexia. All were aged over 18 years. Following good practice guidelines recommended by the British Psychological Society (2017), explicit informed consent was obtained from all participants prior to their completion of the survey, and at the end they were required actively to submit their data for inclusion in the study. Ethical approval was granted by the University Psychology Ethics Committee. In total, 163 respondents completed the survey. Of these, 145 answered either “yes” or “no” to the question “Are you dyslexic? Please only answer yes if you have been formally assessed and told that you are dyslexic”. Data from the remaining 18, who answered “I haven't been assessed but I think I may be”, were removed.

Of the remaining 145 participants, 72 had dyslexia (54 females, aged 18 to 54 years; *M*age = 26.14, *SD* = 8.73) and 73 did not (54 females, aged 19 to 38 years; *M*age = 22.74, *SD* = 4.01). The students with dyslexia had all been formally assessed; in the UK, diagnostic assessments are only carried out by a certified professional who is qualified to assess for specific learning difficulties, so we can be confident that all students identified as having dyslexia met a minimum threshold of difficulties. Although participants were not asked to provide evidence of their assessment, the validity of self‐identification has been previously demonstrated (Jones, Asbjørnsen, Manger, & Eikeland, 2011; Nalavany, Logan, & Carawn, 2018). Participants' age at time of assessment ranged from 6 to 53 years (*M*age = 19.21, *SD* = 10.30). Just over half had been assessed at school (53%: *M*age = 11.89 years, range 6–18 years), the remainder in adulthood (*M*age = 27.38 years, range 19–53 years). The students without dyslexia had no reported literacy difficulties.

In response to a question asking about support received, of those students with dyslexia who had been assessed in childhood, 32% received no support at school, 58% received a little, and 10% received a lot. Within this group, 34% subsequently received no support at university, 37% received a little, and 29% received a lot. Of those who had been assessed in adulthood, 82% received no support at school, 12% received a little, and 6% received a lot; 18% subsequently received no support at university, 62% received a little, and 20% received a lot.

The groups were well‐matched in terms of their favourite school subjects: for students with dyslexia these were maths (17%), drama (14%), science (biology, chemistry, physics: 13%), art (11%), humanities (10%) and languages (English, French: 8%); for students without dyslexia they were science (21%), maths (11%), humanities (10%), languages (English, Norwegian, Spanish: 10%) and art (8%). While no attempt was made to match the groups for subject currently being studied, they proved to be well matched on this: the most frequent degree subjects studied by readers with dyslexia were subjects allied to medicine (midwifery, paramedic science, medical science, nursing: 28%), psychology (16%), education/child development (11%), maths/computing (7%), science subjects (4%), and the arts (including fashion, illustration and design: 4%). Subjects most frequently studied by students without dyslexia were subjects allied to medicine (29%), psychology (26%), science subjects (7%), maths (6%), and the arts (6%).

In relation to university level, 7 participants (6 with dyslexia) were studying for diplomas, 121 were studying at undergraduate level (57 with dyslexia), 14 at master's level (6 with dyslexia) and 3 at doctoral level (all with dyslexia). Participants were British (76%), Norwegian (6%), Polish (3%) and single participants from other countries. All were required to speak English either as a first language or a fluent additional language.

### 2.2 Measures

Participants completed an online survey delivered via Qualtrics (http://www.qualtrics.com), which included demographic questions about sex, age, nationality, language(s) spoken, university course being studied, favourite subject at school, whether they are enjoying university (“no” [1], “sometimes” [2], “yes” [3]), and if they had been assessed and told that they are dyslexic. Participants with dyslexia were asked at what age they were assessed and how much support they received at school and university because of their dyslexia (“none” [0], “a little” [1], “a lot”[2]). All participants then completed the following scales which were selected as established measures of the variables of interest:

*Rosenburg's Self‐Esteem Scale* (Rosenberg, 1965). This widely used measure of global self‐esteem has reported alpha coefficients above .81 across studies, nations and languages, indicating excellent internal consistency (Alessandri, Vecchione, Eisenberg, & Laguna, 2015; Schmitt & Allik, 2005). It consists of 10 items rated on a four point Likert‐type scale from [0] “strongly disagree” to [3] “strongly agree”. Five statements are positively phrased (e.g., “I feel that I have a number of good qualities”) and five are negatively phrased (e.g., “I feel I do not have much to be proud of”). The negatively phrased statements are reverse scored so a higher total score indicates higher self‐esteem, with possible scores from 0 to 30.

*General Self‐Efficacy Scale* (Schwarzer & Jerusalem, 1995). This measure of perceived self‐efficacy assesses optimistic self‐beliefs, explicitly referring to the belief that success results from one's actions. It consists of ten items (e.g., “I can usually handle whatever comes my way”) with responses on a scale from [1] “not at all true” to [4] “exactly true”. Responses are summed to provide an overall score from 10 to 40; the higher the score the more efficacious the person. The scale's validity and reliability (Cronbach's alpha = .86) have been confirmed within numerous research areas (Luszczynska, Scholz, & Schwarzer, 2005).

*Creative Personality Scale* (International Personality Item Pool, 2001). This 20 item scale measures creativity and imagination, and was based on items from the Hogan Personality Inventory (e.g., “I am known for having good ideas”; Hogan & Hogan, 1992) which has an IPIP scale alpha coefficient of 0.83, and from Cattell's Personality Factors Questionnaire (16PF; Cattell, Cattell, & Cattell, 1993; e.g., “I like to get lost in thought”. Cronbach's alpha = .80). Respondents indicate the extent to which items describe them, on a five‐point scale from [1] “very inaccurate” to [5] “very accurate”. Twelve statements are positively phrased and eight are negatively phrased with the negatively worded statements being reverse scored. Total scores range from 20 to 100 with higher scores indicating greater creativity. The calculated Cronbach's alpha for this combined scale is .80.

*Estimated Intelligence Quotient (IQ)* (Furnham & Gasson, 1998). Participants were shown a graph of the normal distribution of the general population's IQ with labels and numbers indicating what each section represents: 70 was labelled “below average”, 100 “Average” and 130 “Superior”. This was accompanied by the instruction: “Look at the following graph showing the distribution of intelligence quotients (IQ scores) across the general population. The average IQ score is 100 and two‐thirds of people's IQ scores fall within the range 85–115. Using this graph as a guide, please estimate your own IQ”. This type of question has been used widely in previous research (e.g., Furnham & Chamorro‐Premuzic, 2005; Kirkcaldy, Noack, Furnham, & Siefen, 2007; Petrides, Furnham, & Martin, 2004). While correlations between estimated and measured IQ are not high – generally in the range *r* = .25 to .50 (Furnham & Chamorro‐Premuzic, 2004) – the aim of using self‐report in the current study was to gauge participants' *perceptions* of their intelligence rather than to assess their actual IQ.

### 2.3 Procedures

Participants completed the measures in the same order: demographic questions followed by the Self‐Esteem Scale, the Self‐Efficacy Scale, the Creative Personality Scale and the Estimated Intelligence Quotient. Finally, participants confirmed their consent to their data being submitted for inclusion in the study. All data were fully completed and submitted by respondents. Analyses were conducted using SPSS version 25 (IBM Corporation, Armonk, NY, USA).

## 3 RESULTS

### 3.1 Enjoyment of university

**FIGURE 1:**
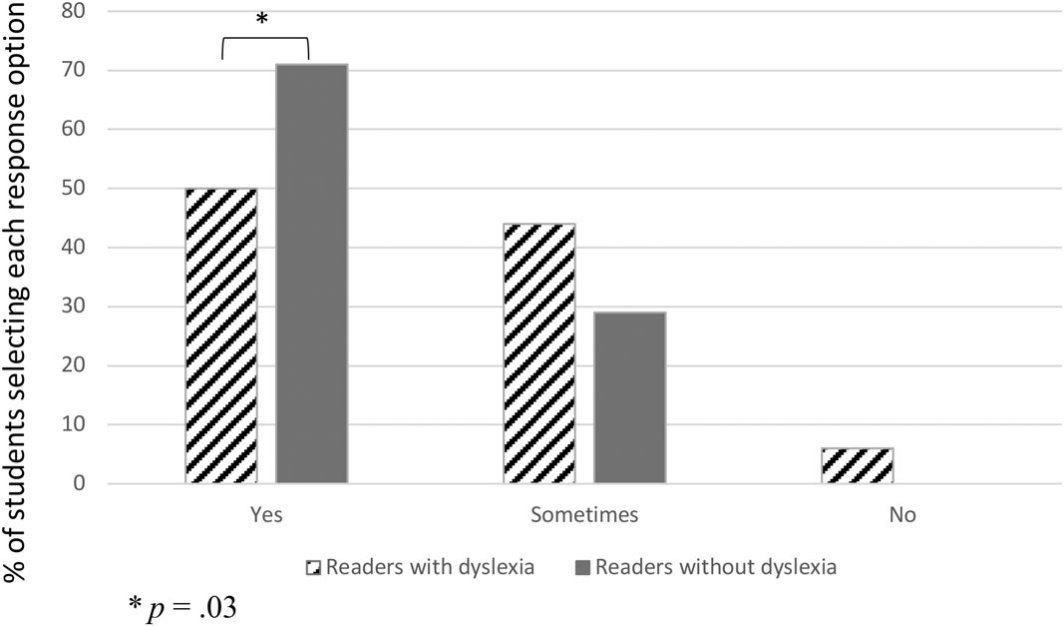
Student responses to the question “are you enjoying university?” by reading ability group

Students with and without dyslexia differed in their reported enjoyment of university (Figure 1): *X* ^2^ (2, *N* = 145) = 9.19, *p* = .01. Only 50% of students with dyslexia were enjoying university, 44% were enjoying it sometimes, and 6% were not enjoying it at all. By contrast, most students without dyslexia were enjoying university (71%), while a few were sometimes enjoying it (29%). Post‐hoc *z*‐tests run on the adjusted residuals revealed that students with dyslexia were significantly less likely than students without dyslexia to be enjoying university (*p* = .03).

### 3.2 Self‐esteem, self‐efficacy, self‐rated creativity and estimated intelligence

**FIGURE 2:**
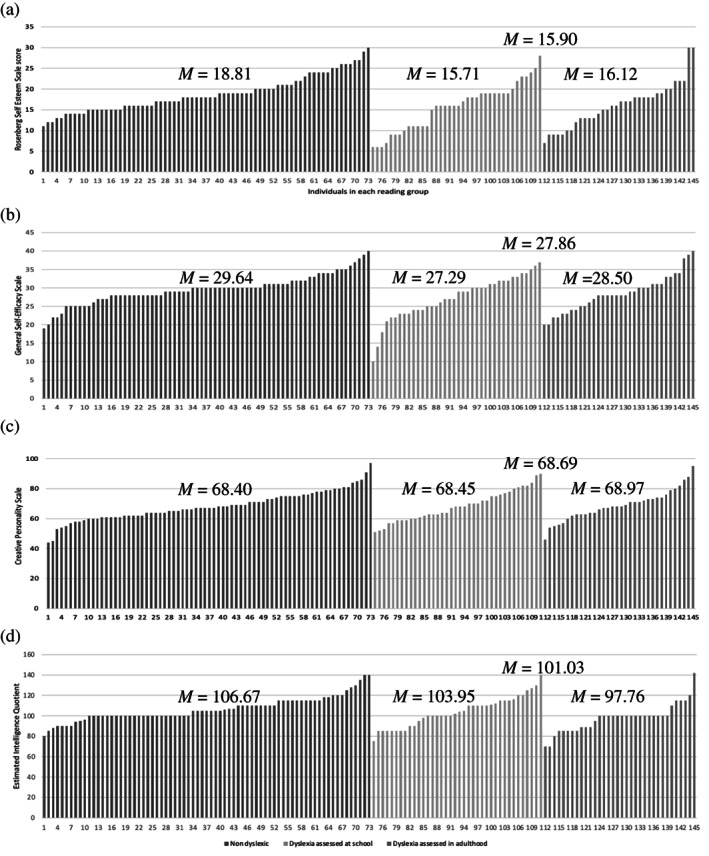
Scores for individual students on (a) self‐esteem; (b) self‐efficacy; (c) creativity; (d) estimated intelligence, sub‐divided by reading group

Both groups had positive self‐esteem, largely within the typical range (15 to 25—see Table 1) although the readers with dyslexia scored towards the lower end of this range and showed greater variability in their scores which lowered their group mean values (Figure 2a). Both groups displayed generally good self‐efficacy around the normative mean value (29.28: Schwarzer, 1993) although the group mean for students with dyslexia was slightly lower than this—again pulled down by a few students with particularly low self‐efficacy scores—and the mean for those without dyslexia was slightly higher (Figure 2b). Self‐rated creativity was comparable across groups (Figure 2c), and estimated intelligence was within one standard deviation around the mean (85–115 IQ points) for both groups. However, within the sample of students with dyslexia there was much greater variability, with many students estimating their intelligence to be below average, while most non‐dyslexic students estimated their intelligence to be average or above average (Figure 2d).

**TABLE 1:** Mean scores by reading ability group, sub‐divided by age of assessment for the readers with dyslexia

	*Measure*			
	Self‐esteem	Self‐efficacy	Creativity	Intelligence
Readers without dyslexia (*n* = 73)				
*Mean (SD)*	18.81 (4.29)	29.64 (3.99)	68.40 (9.66)	106.67 (11.81)
*Quartiles*	15.5, 21	28, 31.5	62, 75	100, 115
Readers with dyslexia (*n* = 72)				
*Mean (SD)*	15.90 (5.55)	27.86 (5.48)	68.69 (10.13)	101.03 (14.73)
*Quartiles*	11, 19	24, 31	62, 75	89, 110
Readers with dyslexia assessed in childhood (*n* = 38)				
*Mean (SD)*	15.71 (5.70)	27.29 (5.88)	68.45 (10.19)	103.95 (14.92)
*Quartiles*	11, 19	23, 32	60, 76	90, 115
Readers with dyslexia assessed in adulthood (*n* = 34)				
*Mean (SD)*	16.12 (5.45)	28.50 (5.00)	68.97 (10.21)	97.76 (14.02)
*Quartiles*	12.8, 19	24.8, 31	63, 74	88, 100

Multivariate ANOVA revealed a statistically significant difference in self‐perception between the readers with and without dyslexia, *F*(4, 14) = 4.08, *p* = .004; Pillai's Trace = .10, $\eta_{P}^{2}$ = .10. Compared to readers without dyslexia, those with dyslexia had significantly lower self‐esteem, *F*(1, 143) = 12.46, *p* < .001, $\eta_{P}^{2}$ = .08, lower self‐efficacy, *F*(1, 143) = 5.03, *p* < .03, $\eta_{P}^{2}$ = .03, and lower estimated intelligence, F(1, 143) = 6.49, *p* = .01, $\eta_{P}^{2}$ = .04. No significant difference was found between the groups in terms of creativity, *F*(1, 143) = 3.20, *p* = .86.

### 3.3 Self‐concept and age of assessment

A second multivariate ANOVA was run to investigate the source of these group differences by dividing the readers with dyslexia into those who were assessed at school and those who were assessed in adulthood, and comparing these groups to the readers without dyslexia (Table 1).

**FIGURE 3:**
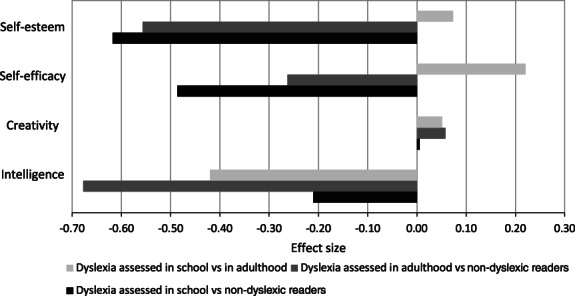
Effect sizes (Z‐scores) showing differences between the two groups of readers with dyslexia and the non‐dyslexic readers on each measure. *Z* scores were derived from the differences between groups using pooled standard deviations

This analysis again revealed a significant main effect of reading ability group, F(8, 280) = 2.92, *p* = .004; Pillai's Trace = .15, $\eta_{P}^{2}$ = .08, with between‐group effects for self‐esteem, *F*(2, 142) = 6.25, *p* = .002, $\eta_{P}^{2}$ = .08; self‐efficacy, *F*(2, 142) = 3.09, *p* < .05, η_p_^2^ = .04, and estimated intelligence, *F*(2, 142) = 5.28, *p* = .006, $\eta_{P}^{2}$ = .07, but no significant difference for self‐rated creativity (*p* > .05)—see Figure 3. Post hoc comparisons using Bonferroni correction indicated that the readers with dyslexia who had been assessed in school had significantly lower self‐esteem than the non‐dyslexic readers (*p* < .01), and also lower self‐efficacy (*p* < .05). The readers with dyslexia who had been assessed in adulthood had significantly lower self‐esteem than the non‐dyslexic readers (*p* = .03) and their estimated intelligence was also significantly lower (*p* = .004).

In terms of estimated intelligence, those students whose dyslexia had been assessed in adulthood were more likely to judge their intelligence to be greater than one standard deviation below the mean (below 85 IQ points: 8.8% of the sample) than greater than one standard deviation above the mean (above 115 IQ points: 5.9%). This is in contrast with those students whose dyslexia was assessed in school (2.6% and 18.4%) and students without dyslexia (1.4% and 15.1%).

### 3.4 Self‐concept and support

**FIGURE 4:**
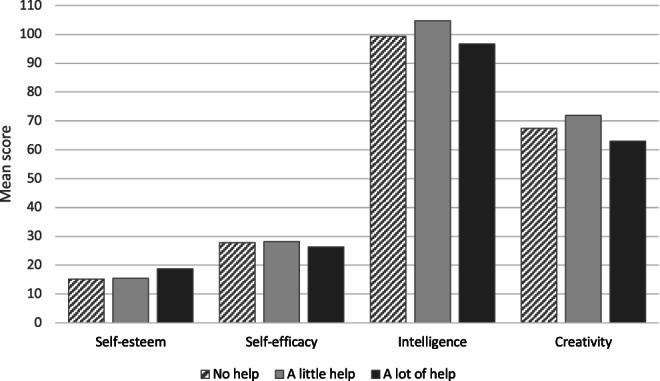
Mean score on each variable according to amount of help received in school, only for the students whose dyslexia was assessed at school

Of the 38 students whose dyslexia had been identified in childhood, only 26 (68%) had received any specific support at school; 22 (58%) reported receiving “a little” support and 6 (10%) reported receiving “a lot” of support. While these numbers preclude statistical analysis, Figure 4 shows the mean score on each variable for students with dyslexia who had received either no support, a little support or a lot of support at school.

### 3.5 Inter‐relationships between the variables in readers with and without dyslexia

A Pearson's Product moment correlation explored relationships between variables across reading groups (Table 2). This revealed significant positive relationships between self‐esteem and self‐efficacy, *r*(145) = .64, *p* < .01, self‐esteem and estimated intelligence, *r*(145) = .25, *p* < .01, and between self‐esteem and enjoyment of university, *r*(145) = .34, *p* < .01. The same pattern emerged for self‐efficacy which correlated positively with self‐rated IQ, *r*(145) = .25, *p* < .01, and enjoyment of university, *r*(145) = .33, *p* < .01. Creativity correlated significantly with estimated intelligence, *r*(145) = .35, *p* < .01.

**TABLE 2:** Pearson Product moment correlations assessing relationships between variables

Measures	1	2	3	4	5
Self‐esteem	–				
2 Self‐efficacy	.64^**^	–			
3 Creativity	−.04	.10	–		
4 Intelligence	.25^**^	.25^**^	.35^**^	–	
5 Enjoyment of university	.34^**^	.33^**^	.07	.15	‐

^**^ *p* < .01.

## 4 DISCUSSION

The current study explored the relationship between self‐esteem, self‐efficacy, estimated intelligence and creativity in university students who either had dyslexia or no literacy difficulties. Furthermore, the students with dyslexia were sub‐divided into those who had been assessed in childhood and those who had been assessed in adulthood to see what effects age of assessment might have on self‐concept. We hypothesized that readers with dyslexia would have lower self‐esteem, lower self‐efficacy and lower estimated intelligence than readers without dyslexia, but higher estimated creativity. An earlier dyslexia assessment was hypothesized to reduce these negative effects on self‐esteem, self‐efficacy and estimated intelligence.

As predicted, students with dyslexia expressed significantly lower self‐esteem and self‐efficacy than students without dyslexia; their estimated intelligence was lower, but there was no significant difference between groups in their reported creativity. This finding of reduced self‐esteem and self‐efficacy amongst university students with dyslexia is consistent with the literature (e.g., Ben‐Naim, Laslo‐Roth, Einav, Biran, & Margalit, 2017; Ingesson, 2007; McIlroy, Poole, Ursavas, & Moriarty, 2015). Similarly, the lower estimated intelligence of the students with dyslexia is in line with reports that students with dyslexia frequently feel less intelligent than their peers, or fear being perceived as less intelligent and lacking academic ability (Hughes & Dawson, 1995; Humphrey & Mullins, 2002; Mortimore & Crozier, 2006). While the estimated intelligence of most students without dyslexia fell within the range of IQs reported in previous studies (around 100–115, Furnham & Mkhize, 2003, Kirkcaldy et al., 2007), or above this, estimates of the students with dyslexia were more likely to be towards the lower end of the range of possible IQ scores. This perception can feed into feelings of negative self‐worth and the perception of poor prospects in life beyond education. As Boetsch, Green, and Pennington (1996) noted, while readers with dyslexia in the workplace may no longer be reminded of their learning difficulties, they still perceive themselves to be less intelligent than their colleagues.

However, when the students with dyslexia were sub‐divided a slightly different picture emerged: those who had been assessed in school had significantly poorer self‐esteem and self‐efficacy than did unimpaired readers but there was no difference in their estimated intelligence. In contrast, those who had been assessed as adults showed significantly lower self‐esteem and estimated intelligence but their self‐efficacy did not differ from that of their non‐dyslexic peers.

The finding that for both groups of students the identification of dyslexia was associated with lower self‐esteem was contrary with expectations. It had been expected that children who were identified as having dyslexia at school, and who received appropriate support, would have a more positive outcome in terms of their psychological well‐being (Lithari, 2019; Riddick, 2000; Wiener & Tardif, 2004). Burden and Burdett (2007) reported that boys attending a specialist dyslexia school held largely positive views about their dyslexia, describing it in surmountable terms: “It's like a lock and key. If you've got enough persistence you can sort of find that key to unlock that door. If you keep doing it, you keep unlocking all the doors, so eventually you get to the end passage.” (p. 79). This may be because of support and understanding provided by specialist teachers; it could also reflect a change in the way children perceive themselves, as Glazzard (2010) noted: “… the diagnosis of dyslexia and the ownership of the label had a positive impact on the students' self‐esteem… They spoke about [it] as a defining moment in… shaping their identity…” (p. 67; also Frederickson & Cline, 2009; McNulty, 2003). Thus, early identification of dyslexia, when young people are forming their self‐concept, can lead to dyslexia being assimilated in a positive way into the self‐identity.

Of course, the key issue may be not the identification of dyslexia, but the provision of appropriate support for the difficulties it brings. If dyslexia is identified *but not supported*, or if it is perceived negatively by teachers and classmates, then the children may feel different to their peers, disempowered and less able to succeed. This may adversely affect their developing self‐concept (Doiku‐Avlidou, 2015). In the current study, of those students with dyslexia who had been assessed in school, approximately two‐thirds reported receiving support (58% a little, 10% a lot) and a third (32%) received no support. These ratings were subjective – we did not quantify ‘a little' or ‘a lot' of support – and sub‐dividing the group in this way resulted in small numbers which precluded statistical analysis. However, it would seem that receiving a lot of support (rather than a little or none) is associated with higher self‐esteem; receiving a little support (rather than none or a lot) is associated with higher estimated intelligence and creativity; receiving some support or none seems to have little effect on self‐efficacy. While the sample size in the current study does not allow definitive conclusions to be drawn, this is an interesting pattern that should be explored further.

It may be that those readers in the current study whose dyslexia was identified in childhood may have displayed more extreme reading difficulties than those who were identified later, hence leading to their lower self‐esteem and self‐efficacy. Future research might objectively test reading ability, and match participants on this to see if these differences remain, however, recent research comparing the literary and cognitive skills of students with early versus late assessed dyslexia indicates that the two groups are highly comparable (Bazen, van den Boer, de Jong, & de Bree, 2020).

It is also possible that global self‐esteem is too broad a concept to use to explore subtle differences in the self‐concept of readers with dyslexia. A meta‐analysis by McArthur et al. (2020) found a stronger significant association between poor (dyslexic) reading and academic self‐concept than between poor reading and global self‐concept (see also Zeleke, 2004). These authors point out that further research should explore the association between poor reading and self‐concept, particularly focussing on academic self‐concept, and particularly with adult poor readers.

Where the students with dyslexia in our study did not show lower self‐efficacy following a late assessment, this may reflect a form of self‐empowerment as they developed creative coping strategies and succeeded in school despite their cognitive and behavioural difficulties. This academic success may have made them more confident in their abilities and protected their self‐efficacy (see Doiku‐Avlidou, 2015; conversely, Carroll & Iles, 2006 found an association between dyslexia assessment in adulthood, ineffective coping strategies and high levels of anxiety, leading to poor self‐perception). It may also be that individuals compensated for their school difficulties by achieving outside of academia, and that these achievements bolstered their self‐confidence and well‐being (Doiku‐Avlidou, 2015; McNulty, 2003).

Interestingly though, while these adults with dyslexia who had been assessed late did not show lower self‐efficacy, their estimates of intelligence were lower; by contrast, those who had been assessed in childhood did not estimate their intelligence significantly lower than their non‐dyslexic peers. This is surprising, as Livingston, Siegel, and Ribary (2018) report: “Children with dyslexia… perceive a stronger relationship between intelligence and reading ability than children without dyslexia and therefore are more likely to feel unintelligent” (p. 10; also Humphrey & Mullins, 2002). Leitão et al. (2017) suggested that negative self‐perceptions in children with dyslexia may be more common prior to assessment, when children attribute their lack of progress to them being “‘lazy’, ‘dumb’ and ‘different’ to their peers” (p. 326). Following an assessment these children may explain their academic difficulties in terms of dyslexia rather than their own intellectual failing. If dyslexia is not identified in childhood but academic difficulties continue, then individuals are more likely to question their intellectual ability, leading to lower estimated IQ amongst readers with dyslexia who are assessed as adults (Gibson & Kendall, 2010; Livingston et al., 2018; McNulty, 2003). According to Armstrong and Humphrey (2009), a dyslexia assessment in late adolescence or adulthood, by which time self‐identity is largely fixed, is more likely to result in a reduction in academic motivation and outcomes.

Contrary with predictions, no overall between‐group differences were found for creativity. There are a number of possible explanations for this. While the present study assessed creativity using a survey, it may be that had it been measured objectively, differences would have been observed. A similar argument has been made regarding paper‐and‐pencil measures of visuospatial ability (von Károlyi, Winner, Gray, & Sherman, 2003; also see Ritchie et al. [2013] for a discussion of validity in creativity research). In addition to this, as Wolff and Lundberg (2002) reported, university students with dyslexia often study art subjects, possibly due to high creativity or avoidance of the “written” subject (see also Bacon & Bennett, 2013). This was not the case in the current study in which most participants (in both groups) were studying science subjects, maybe adding to their lower perceptions of their own creativity. Finally, a recent meta‐analysis of dyslexia and creativity found that relative to adults in the general population, adults with dyslexia show greater performance variability in non‐verbal creativity (but smaller variability in verbal creativity: Erbeli et al., 2022). Similarly, Chamberlain et al's (2018) meta‐analysis of dyslexia and visuospatial ability (creative differences between readers with and without dyslexia are mostly reported in the visuospatial domain: Bacon & Bennett, 2013; Tafti et al., 2009), found that readers with dyslexia showed greater performance variability. In comparison to readers without dyslexia they were significantly more likely to perform either extremely poorly or extremely well (Chamberlain, Brunswick, Siev, & McManus, 2018). The distribution of scores in the present study, across all measures, reflected this pattern, although the extent to which this is a factor of the sample size remains to be determined.

In view of changes in legislation over the last two decades to identify and support the needs of readers with dyslexia in the UK (e.g., the Special Educational Needs and Disability Code of Practice: 0–25 years; Department for Education, 2014), it is surprising that so many students with dyslexia in the current study were assessed in adulthood rather than childhood. However, recent figures indicate that over 80% of children with dyslexia are not assessed at school (All‐Party Parliamentary Group on Dyslexia and Other Specific Learning Difficulties, 2019b), and many may only be assessed once they reach university (Nichols, McLeod, Holder, & McLeod, 2009). Thus it is important to determine the potential psychological impacts of age of assessment so university students with dyslexia can be supported appropriately, not only in terms of their academic needs but also their self‐perceptions, so they may develop the emotional resilience necessary to enable them to succeed in higher education and beyond.

It must be reiterated that this study is limited by its sample size and the fact that participants were all university students, so they have been more successful in their academic studies – and may come from the less severe end of the dyslexia distribution – than many other readers with dyslexia. Nevertheless these are important preliminary findings that warrant further investigation. Future studies might test the reliability of these findings in a broader sample including non‐university students (with assessed reading ability), focussing on different areas of self‐esteem and self‐efficacy, and the relationship between estimated intelligence and measured intelligence. They might also consider estimated versus measured multiple intelligences in readers with and without dyslexia to determine the extent to which students' perceptions of their strengths and difficulties reflect their true abilities, and the academic and emotional benefits that such insight might offer.

In summary, this study has identified some important differences in the self‐perceptions of students without dyslexia and with dyslexia that was assessed either in childhood or adulthood. An early assessment was associated with lower self‐esteem and self‐efficacy but no difference in estimated intelligence, while a late assessment was associated with lower self‐esteem and estimated intelligence but no difference in self‐efficacy. No between‐group differences were found for creativity. Possible reasons for these findings have been considered and future avenues for research have been proposed.

## CONFLICT OF INTEREST

None.

## Data Availability

The data that support the findings of this study are available at: https://osf.io/mwnb3/

## References

[dys1722-bib-0001] Alessandri, G. , Vecchione, M. , Eisenberg, N. , & Laguna, M. (2015). On the factor structure of the Rosenberg (1965) General Self‐Esteem Scale. Psychological Assessment, 27, 621–635. 10.1037/pas0000073 25580614

[dys1722-bib-0002] All‐Party Parliamentary Group for Dyslexia and Other Specific Learning Difficulties . (2019a). The human cost of dyslexia. London: All Party Parliamentary Group on Specific Learning Difficulties. Retrieved from: http://bit.ly/APPG-human

[dys1722-bib-0003] All‐Party Parliamentary Group for Dyslexia and Other Specific Learning Difficulties . (2019b). Educational cost of dyslexia. London: All Party Parliamentary Group on Specific Learning Difficulties. Retrieved from: http://bit.ly/appg-educational

[dys1722-bib-0004] Alves, R. , & Nakano, T. (2014). Creativity and intelligence in children with and without developmental dyslexia. Paidéia (Ribeirão Preto), 24, 361–369. 10.1590/1982-43272459201410

[dys1722-bib-0005] Arens, A. K. , & Jansen, M. (2016). Self‐concepts in reading, writing, listening, and speaking: A multidimensional and hierarchical structure and its generalizability across native and foreign languages. Journal of Educational Psychology, 108(5), 646–664. 10.1037/edu0000081

[dys1722-bib-0006] Armstrong, D. , & Humphrey, N. (2009). Reactions to a diagnosis of dyslexia among students entering further education: Development of the ‘resistance–accommodation' model. British Journal of Special Education, 36, 95–102. 10.1111/j.1467-8578.2008.00408.x

[dys1722-bib-0007] Bacon, A. M. , & Bennett, S. (2013). Dyslexia in higher education: The decision to study art. European Journal of Special Needs Education, 28, 19–32. 10.1080/08856257.2012.742748

[dys1722-bib-0008] Bandura, A. (2010). Self‐efficacy. In The Corsini Encyclopedia of Psychology (pp. 1–3). Hoboken, NJ: John Wiley and Sons Inc. 10.1002/9780470479216.corpsy0836

[dys1722-bib-0009] Bazen, L. , van den Boer, M. , de Jong, P. F. , & de Bree, E. H. (2020). Early and late diagnosed dyslexia in secondary school: Performance on literacy skills and cognitive correlates. Dyslexia, 26, 359–376. 10.1002/dys.1652 31994792 PMC7687086

[dys1722-bib-0010] Ben‐Naim, S. , Laslo‐Roth, R. , Einav, M. , Biran, H. , & Margalit, M. (2017). Academic self‐efficacy, sense of coherence, hope and tiredness among college students with learning disabilities. European Journal of Special Needs Education, 32, 18–34. 10.1080/08856257.2016.1254973

[dys1722-bib-0011] Boetsch, E. A. , Green, P. A. , & Pennington, B. F. (1996). Psychosocial correlates of dyslexia across the life span. Development and Psychopathology, 8, 539–562. 10.1017/S0954579400007264

[dys1722-bib-0012] British Psychological Society . (2017). Ethics guidelines for internet‐mediated research. INF206/04.2017. Leicester: Author. Retrieved from: https://www.bps.org.uk/news-and-policy/ethics-guidelines-internet-mediated-research-2017

[dys1722-bib-0013] Brown, J. D. , & Marshall, M. A. (2006). The three faces of self‐esteem. In M. H. Kernis (Ed.), Self‐esteem Issues and Answers: A Sourcebook of Current Perspectives (pp. 4–9). Hove: Psychology Press.

[dys1722-bib-0014] Brunswick, N. (2009). Dyslexia: A Beginner's Guide. Oxford: Oneworld Publications.

[dys1722-bib-0015] Burden, R. (2008). Is dyslexia necessarily associated with negative feelings of self‐worth? A review and implications for future research. Dyslexia, 14, 188–196. 10.1002/dys.371 18646275

[dys1722-bib-0016] Burden, R. , & Burdett, J. (2007). What's in a name? Students with dyslexia: Their use of metaphor in making sense of their disability. British Journal of Special Education, 34, 77–82. 10.1111/j.1467-8578.2007.00459.x

[dys1722-bib-0017] Burns, E. , Poikkeus, M. , & Aro, M. (2013). Resilience strategies employed by teachers with dyslexia working at tertiary education. Teaching and Teacher Education, 34, 77–85. 10.1016/j.tate.2013.04.007

[dys1722-bib-0018] Carroll, J. M. , & Iles, J. E. (2006). An assessment of anxiety levels in dyslexic students in higher education. British Journal of Educational Psychology, 76, 651–662. 10.1348/000709905X66233 16953967

[dys1722-bib-0019] Cattell, R. B. , Cattell, A. K. , & Cattell, H. E. (1993). Sixteen Factor Questionnaire (5th ed.). Champaign, IL: Institute for Personality and Ability Testing.

[dys1722-bib-0020] Chamberlain, R. , Brunswick, N. , Siev, J. , & McManus, I. C. (2018). Meta‐analytic findings reveal lower means but higher variances in visuospatial ability in dyslexia. British Journal of Psychology, 109, 897–916. 10.1111/bjop.12321 29938776

[dys1722-bib-0021] Cockcroft, K. , & Hartgill, M. (2004). Focusing on the abilities in learning disabilities: Dyslexia and creativity. Education as Change, 8, 61–79. 10.1080/16823200409487081

[dys1722-bib-0022] de Beer, J. , Engels, J. , Heerkens, Y. , & van der Klink, J. (2014). Factors influencing work participation of adults with developmental dyslexia: A systematic review. BMC Public Health, 14, 77. 10.1186/1471-2458-14-77 24460949 PMC3913008

[dys1722-bib-0023] Department for Education (2014). Special Educational Needs and Disability Code of Practice: 0–25 years. London: HMSO.

[dys1722-bib-0024] Doiku‐Avlidou, M. (2015). The educational, social and emotional experiences of students with dyslexia: The perspective of postsecondary education students. International Journal of Special Education, 30, 132–145. https://eric.ed.gov/?id=EJ1094794

[dys1722-bib-0025] Erbeli, F. , Peng, P. , & Rice, M. (2022). No evidence of creative benefit accompanying dyslexia: A meta‐analysis. Journal of Learning Disabilities, 55(3), 242–253. 10.1177/00222194211010350 33899570

[dys1722-bib-0026] Firth, N. , Frydenberg, E. , Steeg, C. , & Bond, L. (2013). Coping successfully with dyslexia: An initial study of an inclusive school‐based resilience programme. Dyslexia, 19, 113–130. 10.1002/dys.1453 23526752

[dys1722-bib-0027] Frederickson, N. , & Cline, T. (2009). Special Educational Needs, Inclusion and Diversity (2nd ed.). Maidenhead, England: Open University Press.

[dys1722-bib-0028] Furnham, A. , & Chamorro‐Premuzic, T. (2004). Estimating one's own personality and intelligence scores. British Journal of Psychology, 95, 1–12. 10.1348/000712604773952395 15142299

[dys1722-bib-0029] Furnham, A. , & Chamorro‐Premuzic, T. (2005). Estimating one's own and one's relatives' multiple intelligence: A study from Argentina. The Spanish Journal of Psychology, 8, 12–20. 10.1017/S1138741600004911 15875453

[dys1722-bib-0030] Furnham, A. , & Gasson, L. (1998). Sex differences in parental estimates of their children's intelligence. Sex Roles, 38, 151–162. 10.1023/A:1018772830511

[dys1722-bib-0031] Furnham, A. , & Mkhize, N. (2003). Zulu mothers' beliefs about their own and their children's intelligence. The Journal of Social Psychology, 143, 83–94. 10.1080/00224540309598432 12617348

[dys1722-bib-0032] Gerber, P. J. , Ginsberg, R. , & Reiff, H. B. (1992). Identifying alterable patterns in employment success for highly successful adults with learning disabilities. Journal of Learning Disabilities, 25, 475–487. 10.1177/002221949202500802 1460391

[dys1722-bib-0033] Gibson, S. , & Kendall, L. (2010). Stories from school: Dyslexia and learners' voices on factors impacting on achievement. Support for Learning, 25, 187–193. 10.1111/j.1467-9604.2010.01465.x

[dys1722-bib-0034] Glazzard, J. (2010). The impact of dyslexia on pupils' self‐esteem. Support for Learning, 25, 63–69. 10.1111/j.1467-9604.2010.01442.x

[dys1722-bib-0035] Hellendoorn, J. , & Ruijssenaars, W. (2000). Personal experiences and adjustment of Dutch adults with dyslexia. Remedial and Special Education, 21, 227–239. 10.1177/074193250002100405

[dys1722-bib-0036] Higher Education Statistics Agency . (2014). https://www.hesa.ac.uk

[dys1722-bib-0037] Hogan, R. , & Hogan, J. (1992). Hogan Personality Inventory Manual. Tulsa, OK: Hogan Assessment Systems.

[dys1722-bib-0038] Hughes, W. , & Dawson, R. O. N. (1995). Memories of school: Adult dyslexics recall their school days. Support for Learning, 10, 181–184. 10.1111/j.1467-9604.1995.tb00037.x

[dys1722-bib-0039] Humphrey, N. , & Mullins, P. M. (2002). Self‐concept and self‐esteem in developmental dyslexia. Journal of Research in Special Educational Needs, 2(2), 1–13. 10.1111/j.1471-3802.2002.00163.x

[dys1722-bib-0040] Ingesson, S. G. (2007). Growing up with dyslexia: Interviews with teenagers and young adults. School Psychology International, 28, 574–591. 10.1177/0143034307085659

[dys1722-bib-0041] International Personality Item Pool . (2001). A Scientific Collaboratory for the Development of Advanced Measures of Personality Traits and Other Individual Differences. https://ipip.ori.org/

[dys1722-bib-0042] Jones, L. O. , Asbjørnsen, A. , Manger, T. , & Eikeland, O. J. (2011). An examination of the relationship between self‐reported and measured reading and spelling skills among incarcerated adults in Norway. The Journal of Correctional Education, 62, 26–50. http://www.jstor.org/stable/23282820

[dys1722-bib-0043] Jones, R. S. P. , & Heskin, K. J. (2010). Self‐concept and learning disability: An experimental analysis. Insights on Learning Disability, 7, 49–55.

[dys1722-bib-0044] Kapoula, Z. , Ruiz, S. , Spector, L. , Mocorovi, M. , Gaertner, C. , Quilici, C. , & Vernet, M. (2016). Education influences creativity in dyslexic and non dyslexic children and teenagers. PLoS One, 11(3), e0150421. 10.1371/journal.pone.0150421 26950067 PMC4780733

[dys1722-bib-0045] Kirkcaldy, B. , Noack, P. , Furnham, A. , & Siefen, G. (2007). Parental estimates of their own and their children's intelligence. European Psychologist, 12, 173–180. 10.1027/1016-9040.12.3.173

[dys1722-bib-0046] Lawrence, D. (1996). Enhancing Self‐Esteem in the Classroom (2nd ed.). London, England: PCP Ltd.

[dys1722-bib-0047] Leitão, S. , Dzidic, P. , Claessen, M. , Gordon, J. , Howard, K. , Nayton, M. , & Boyes, M. E. (2017). Exploring the impact of living with dyslexia: The perspectives of children and their parents. International Journal of Speech‐Language Pathology, 19, 322–334. 10.1080/17549507.2017.1309068 28394222

[dys1722-bib-0048] Lithari, E. (2019). Fractured academic identities: Dyslexia, secondary education, self‐esteem and school experiences. International Journal of Inclusive Education, 23, 280–296. 10.1080/13603116.2018.1433242

[dys1722-bib-0049] Livingston, E. M. , Siegel, L. S. , & Ribary, U. (2018). Developmental dyslexia: Emotional impact and consequences. Australian Journal of Learning Difficulties, 23, 107–135. 10.1080/19404158.2018.1479975

[dys1722-bib-0050] Łockiewicz, M. , Bogdanowicz, K. M. , & Bogdanowicz, M. (2014). Psychological resources of adults with developmental dyslexia. Journal of Learning Disabilities, 47, 543–555. 10.1177/0022219413478663 23462191

[dys1722-bib-0051] Luszczynska, A. , Scholz, U. , & Schwarzer, R. (2005). The General Self‐Efficacy Scale: Multicultural validation studies. The Journal of Psychology, 139, 439–457. 10.3200/JRLP.139.5.439-457 16285214

[dys1722-bib-0052] Majeed, N. M. , Hartanto, A. , & Tan, J. J. X. (2021). Developmental dyslexia and creativity: A meta‐analysis. Dyslexia, 27, 187–203. 10.1002/dys.1677 33586314

[dys1722-bib-0053] Marsh, H. W. , & Craven, R. G. (2006). Reciprocal effects of self‐concept and performance from a multidimensional perspective: Beyond seductive pleasure and unidimensional perspectives. Perspectives on Psychological Science, 1, 133–163. 10.1111/j.1745-6916.2006.00010.x 26151468

[dys1722-bib-0054] Marsh, H. W. , & Martin, A. J. (2011). Academic self‐concept and academic achievement: Relations and causal ordering. British Journal of Educational Psychology, 81, 59–77. 10.1348/000709910X503501 21391964

[dys1722-bib-0055] Marsh, H. W. , & O'Mara, A. (2008). Reciprocal effects between academic self‐concept, self‐esteem, achievement, and attainment over seven adolescent years: Unidimensional and multidimensional perspectives of self‐concept. Personality and Social Psychology Bulletin, 34, 542–552. 10.1177/0146167207312313 18340036

[dys1722-bib-0056] McArthur, G. M. , Filardi, N. , Francis, D. A. , Boyes, M. E. , & Badcock, N. A. (2020). Self‐concept in poor readers: A systematic review and meta‐analysis. PeerJ, 8, e8772. 10.7717/peerj.8772 32211239 PMC7081778

[dys1722-bib-0057] McIlroy, D. , Poole, K. , Ursavas, O. F. , & Moriarty, A. (2015). Distal and proximal associates of academic performance at secondary level: A mediation model of personality and self‐efficacy. Learning and Individual Differences, 38, 1–9. 10.1016/j.lindif.2015.01.004

[dys1722-bib-0058] McNulty, M. A. (2003). Dyslexia and the life course. Journal of Learning Disabilities, 36, 363–381. 10.1177/00222194030360040701 15490908

[dys1722-bib-0059] Morgan, P. L. , Fuchs, D. , Compton, D. L. , Cordray, D. S. , & Fuchs, L. S. (2008). Does early reading failure decrease children's reading motivation? Journal of Learning Disabilities, 41, 387–404. 10.1177/0022219408321112 18768772

[dys1722-bib-0060] Mortimore, T. , & Crozier, W. R. (2006). Dyslexia and difficulties with study skills in higher education. Studies in Higher Education, 31, 235–251. 10.1080/03075070600572173

[dys1722-bib-0061] Mourgues, C. V. , Preiss, D. D. , & Grigorenko, E. L. (2014). Reading skills, creativity, and insight: Exploring the connections. The Spanish Journal of Psychology, 17, E58. 10.1017/sjp.2014.59 26055787

[dys1722-bib-0062] Nalavany, B. A. , & Carawan, L. W. (2012). Perceived family support and self‐esteem: The mediational role of emotional experience in adults with dyslexia. Dyslexia, 18, 58–74. 10.1002/dys.1433 22190477

[dys1722-bib-0063] Nalavany, B. A. , Carawan, L. W. , & Brown, L. J. (2011). Considering the role of traditional and specialist schools: Do school experiences impact the emotional well‐being and self‐esteem of adults with dyslexia? British Journal of Special Education, 38, 191–200. 10.1111/j.1467-8578.2011.00523.x

[dys1722-bib-0064] Nalavany, B. A. , Logan, J. M. , & Carawan, L. W. (2018). The relationship between emotional experience with dyslexia and work self‐efficacy among adults with dyslexia. Dyslexia, 24, 17–32. 10.1002/dys.1575 29230916

[dys1722-bib-0066] Nichols, S. A. , McLeod, J. S. , Holder, R. L. , & McLeod, H. S. T. (2009). Screening for dyslexia, dyspraxia and Meares‐Irlen syndrome in Higher Education. Dyslexia, 15, 42–60. 10.1002/dys.382 19089876

[dys1722-bib-0067] Olofsson, Å. , Taube, K. , & Ahl, A. (2015). Academic achievement of university students with dyslexia. Dyslexia, 21, 338–349. 10.1002/dys.1517 26459832

[dys1722-bib-0068] Pąchalska, M. , Bogdanowicz, K. , Tomaszewska, K. , Łockiewicz, M. , & Bogdanowicz, M. (2009). The stimulation of creative activity in dyslexic adults. Acta Neuropsychologica, 7, 113–130.

[dys1722-bib-0069] Petrides, K. V. , Furnham, A. , & Martin, G. N. (2004). Estimates of emotional and psychometric intelligence: Evidence for gender‐based stereotypes. The Journal of Social Psychology, 144, 149–162. 10.3200/SOCP.144.2.149-162 15074503

[dys1722-bib-0070] Pitt, S. , & Soni, A. (2017). Students' experiences of academic success with dyslexia: A call for alternative intervention. Support for Learning, 32, 387–405. 10.1111/1467-9604.12182

[dys1722-bib-0071] Richardson, J. T. E. (2015). Academic attainment in students with dyslexia in distance education. Dyslexia, 21, 323–337. 10.1002/dys.1502 26059744

[dys1722-bib-0072] Riddick, B. (2000). An examination of the relationship between labelling and stigmatisation with special reference to dyslexia. Disability & Society, 15, 653–667. 10.1080/09687590050058233

[dys1722-bib-0073] Ritchie, S. J. , Luciano, M. , Hansell, N. K. , Wright, M. J. , & Bates, T. C. (2013). The relationship of reading ability to creativity: Positive, not negative associations. Learning and Individual Differences, 26, 171. 10.1016/j.lindif.2013.02.009

[dys1722-bib-0074] Rose, J. (2009). Identifying and teaching children and young people with dyslexia and literacy difficulties: An independent report. Department for Children, Schools and Families.

[dys1722-bib-0075] Rosenberg, M. (1965). Society and the Adolescent Self‐Image. Princeton, NJ: Princeton University Press.

[dys1722-bib-0076] Rowan, L. (2014). University transition experiences of four students with dyslexia in New Zealand. Australian Journal of Learning Difficulties, 19, 129–136. 10.1080/19404158.2014.923478

[dys1722-bib-0077] Schmitt, D. P. , & Allik, J. (2005). Simultaneous administration of the Rosenberg Self‐Esteem Scale in 53 nations: Exploring the universal and culture‐specific features of global self‐esteem. Journal of Personality and Social Psychology, 89, 623–642. 10.1037/0022-3514.89.4.623 16287423

[dys1722-bib-0078] Schwarzer, R. (1993). Measurement of Perceived Self‐Efficacy. Psychometric Scales for Cross‐Cultural Research. Berlin, Germany: Freie Universität Berlin.

[dys1722-bib-0079] Schwarzer, R. , & Jerusalem, M. (1995). Generalized Self‐Efficacy Scale. In J. Weinman , S. Wright , & M. Johnston (Eds.), Measures in Health Psychology: A User's Portfolio (pp. 35–37). Windsor, UK: NFER‐NELSON.

[dys1722-bib-0080] Singer, E. (2005). The strategies adopted by Dutch children with dyslexia to maintain their self‐esteem when teased at school. Journal of Learning Disabilities, 38, 411–423. 10.1177/00222194050380050401 16329442

[dys1722-bib-0081] Stampoltzis, A. , Antonopoulou, E. , Zenakou, E. , & Kouvava, S. (2010). Learning sensory modalities and educational characteristics of Greek dyslexic and non‐dyslexic university students. Electronic Journal of Research in Educational Psychology, 8, 561–580. 10.25115/ejrep.v8i21.1383

[dys1722-bib-0082] Stampoltzis, A. , & Polychronopoulou, S. (2009). Greek university students with dyslexia: An interview study. European Journal of Special Needs Education, 24, 307–321. 10.1080/08856250903020195

[dys1722-bib-0083] Tafti, M. A. , Hameedy, M. A. , & Baghal, N. M. (2009). Dyslexia, a deficit or a difference: Comparing the creativity and memory skills of dyslexic and nondyslexic students in Iran. Social Behavior and Personality: An International Journal, 37, 1009–1016. 10.2224/sbp.2009.37.8.1009

[dys1722-bib-0084] von Károlyi, C. , Winner, E. , Gray, W. , & Sherman, G. F. (2003). Dyslexia linked to talent: Global visual–spatial ability. Brain and Language, 85, 427–431. 10.1016/S0093-934X(03)00052-X 12744954

[dys1722-bib-0085] Wiener, J. , & Tardif, C. Y. (2004). Social and emotional functioning of children with learning disabilities: Does special education placement make a difference? Learning Disabilities Research & Practice, 19, 20–32. 10.1111/j.1540-5826.2004.00086.x

[dys1722-bib-0086] Wolff, U. , & Lundberg, I. (2002). The prevalence of dyslexia among art students. Dyslexia, 8, 34–42. 10.1002/dys.211 11990223

[dys1722-bib-0087] Zeigler‐Hill, V. (2013). Self‐Esteem. New York, NY: Psychology Press.

[dys1722-bib-0088] Zeleke, S. (2004). Self‐concepts of students with learning disabilities and their normally achieving peers: A review. European Journal of Special Needs Education, 19, 145–170. 10.1080/08856250410001678469

